# PSYCHOMETRIC PROPERTIES OF A NOVEL MEASURE OF FINANCIAL ABUSE OF OLDER ADULTS

**DOI:** 10.1093/geroni/igac059.2294

**Published:** 2022-12-20

**Authors:** David Hancock, Karl Pillemer, David Burnes, Sara Czaja, Mark Lachs

**Affiliations:** Weill Cornell Medicine, New York City, New York, United States; Cornell University, Ithaca, New York, United States; University of Toronto, Toronto, Ontario, Canada; Weill Cornell Medicine, New York City, New York, United States; Weill Cornell Medicine, New York, New York, United States

## Abstract

**Background and Objectives:**

Elder abuse, or mistreatment affects at least 1 in 10 older adults. Financial abuse, or exploitation, of older adults is among the most commonly reported forms of abuse. Few validated measures exist to measure this construct. We aim to present a new psychometrically validated measure of financial abuse of older adults.Research Design and

**Methods:**

Classical test theory and item response theory methodologies were used to examine a five-item measure of financial abuse of older adults, administered as part of the New York State Elder Mistreatment Survey.

**Results:**

Factor analysis revealed a single factor best fits the data, which we labeled as financial abuse. Moreover, IRT analyses revealed that these items discriminated well between abused and non-abuse persons and provided information at high levels of the latent trait θ, as is expected in cases of abuse. Discussion and Implications: The FIVE has acceptable psychometric properties and has been used successfully in large scale survey research. We recommend this measure as an indicator of financial abuse in elder abuse, or mistreatment prevalence research studies.

